# High Level of Physical Activity Reduces the Risk of Renal Progression in Hypertensive Patients

**DOI:** 10.3390/ijerph17051669

**Published:** 2020-03-04

**Authors:** Po-Ya Chang, Shu-Yu Lyu, Yuh-Feng Lin, Chun-Ching Huang

**Affiliations:** 1 Department of Leisure Industry and Health Promotion, National Taipei University of Nursing and Health Sciences, Taipei City 11219, Taiwan; pychang@ntunhs.edu.tw (P.-Y.C.); sylyu2016@ntunhs.edu.tw (S.-Y.L.); 2 Graduate Institute of Clinical Medicine, Taipei Medical University, Taipei City 11031, Taiwan; linyf@shh.org.tw; 3 Department of Exercise and Health Science, National Taipei University of Nursing and Health Sciences, Taipei City 11219, Taiwan

**Keywords:** physical activity, renal progression, hypertension

## Abstract

Physical activity has long been associated with chronic diseases. However, the association between physical activity and renal progression in hypertensive patients remains unclear. This study investigated the relationship between the level of physical activity and renal function in hypertensive patients. We analyzed 3543 patients with hypertension. Data on patients’ demographic characteristics, comorbidities, physical activity, and lifestyle characteristics were collected via questionnaires. An estimated glomerular filtration rate (eGFR) that was reduced by more than 25% from the baseline eGFR was defined as renal progression. This study divided physical activity into three levels (low, moderate, and high) based on their metabolic equivalent of tasks (METs) levels. The mean age was 63.32 ± 12.29 years. After we adjusted for covariates, renal progression was significantly higher among patients with low levels of physical activity (odds ratio (OR), 1.39; 95% confidence interval (CI), 1.01–1.90)) and moderate levels of physical activity (OR, 1.39; 95% CI, 1.04–1.86) than among patients with high levels of physical activity. We found a significant association between physical activity and renal progression in hypertensive patients, especially in elderly patients and men. Therefore, to reduce the risk of renal progression, we recommend that clinicians should encourage patients to improve their physical activity.

## 1. Introduction

Hypertension is a tremendous global public health threat. It contributes to the burden of coronary artery disease, heart attack, and stroke, as well as chronic kidney disease (CKD) and end-stage renal disease (ESRD) [1,2]. Hypertension is closely related to renal progression and nondiabetic CKD [3,4]. According to the United States Renal Data System (USRDS) reports, 28% of newly diagnosed ESRD is the result of hypertension [5]. It has been reported that the prevalence of ESRD in Taiwan is the highest in the world [5]. In Taiwan, the three most common comorbidities among ESRD patients were hypertension, cardiovascular disease, and diabetes, which were present in 82%, 56.2%, and 49.3%, respectively [6]. Patients with hypertension gradually develop ischemic glomeruli owing to vascular injury [7]. Hypertension that is inadequately or inappropriately treated could progress rapidly to renal failure [7]. In summary, patients with hypertension may experience a decline in renal function and develop renal dysfunction throughout their lives.

Previous studies have proposed that physical exercise has a protective effect against a number of chronic diseases [8,9]. According to World Health Organization (WHO) recommendations, adults should perform at least 150 min of moderate-intensity aerobic physical activity per week, at least 75 min of vigorous-intensity aerobic physical activity per week, or an equivalent combination of moderate- and vigorous-intensity activity [10]. Previous meta-analyses showed that people who reached an overall level of physical exercise that was several times higher than the currently recommended minimum amount of exercise had significantly lower risks of breast cancer, colon cancer, diabetes, ischemic heart disease, and ischemic stroke events [8]. The Kidney Disease Outcomes Quality Initiative (K/DOQI) Clinical Practice Guidelines suggest that increased physical function and a higher level of physical activity could reduce the risk of cardiovascular disease in dialysis patients [11]. Additional benefits may improve patents’ ability to perform basic activities of daily living and quality of life [12]. Among patients with diabetes, not only leisure-time physical activity, but also daily commuting and occupational activity can reduce the risk of cardiovascular disease mortality [13]. Non-vigorous activity as well as vigorous physical activity was associated with insulin sensitivity [14].

Despite the causal relationships between physical inactivity and chronic diseases [15], there is a lack of data regarding the association between the risk of renal progression in hypertensive patients and the level of total physical activity. Therefore, we examined the relationship between the level of physical activity and changes in renal function in hypertensive patients. This analysis will help future interventions to prevent renal progression in high-risk populations.

## 2. Materials and Methods

### 2.1. Study Population

**Figure 1:**
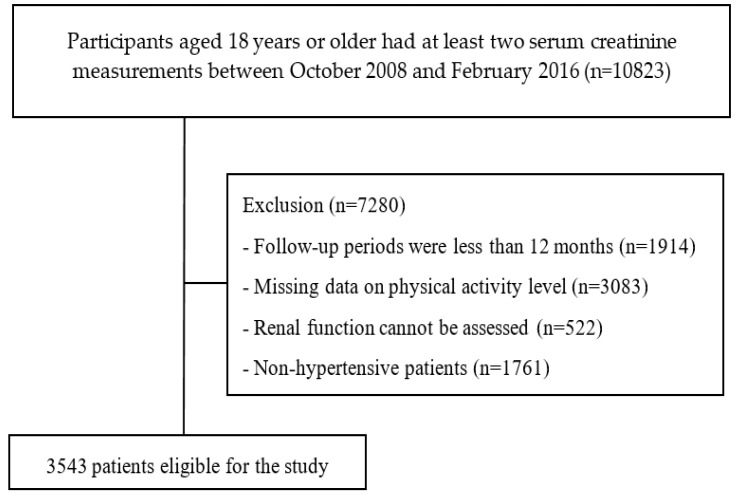
Flowchart of participant analyzed in this study.

The study data were obtained from the Epidemiology and Risk Factors Surveillance of CKD database (2008–2016); these data were collected at 14 medical centers and communities in Taiwan from October 2008 to February 2016. In this study, we identified data from at least two serum creatinine measurements in patients aged ≥18 years, with a total of 10,823 participants. Patients were excluded from the analysis based on the following criteria: follow-up periods were less than 12 months, missing data on the level of physical activity, and inability to assess renal function. Our study used a self-report questionnaire to define patients with hypertension. The diagnosis question was “Have you ever been diagnosed with hypertension by a doctor”? (Answer: “Yes/No”). If the patient answered yes, the patient was defined as having hypertension. In total, 3543 patients with hypertension were enrolled; Figure 1.

### 2.2. Study Variables and Definitions

Data on patients’ demographic characteristics, comorbidities, physical activity, and lifestyle were collected via self-report questionnaires, including sex, age, hypertension, CKD, diabetes mellitus, dyslipidemia, stroke, gout, cigarette smoking, alcohol consumption, sitting time, and levels of physical activity. Patients’ physical examination data were obtained via clinical chart review, including serum creatinine, baseline estimated glomerular filtration rate (eGFR), height, weight, body mass index (BMI), fasting glucose, systolic blood pressure (SBP), diastolic blood pressure (DBP), and total cholesterol.

The definition of CKD was determined according to the Kidney Disease Outcomes Quality Initiative guidelines [16]. The eGFR was calculated using the Chronic Kidney Epidemiology Collaboration (CKD-EPI) The Taiwan equation is as follows: eGFR (mL/min/1.73 m^2^) = 1.262 × (141 × min (serum creatinine (SCr)/κ, 1)^α^ × max (SCr/κ, 1)^−1.209^ × 0.993^age^ × 1.018 (if female) × 1.159 (if black))^0.914^, κ = 0.7 (for female) and 0.9 (for male), α = −0.329 (female), and −0.411 (male); min denotes the minimum of SCr/κ or 1, and max denotes the maximum of SCr/κ or 1 [17].

An eGFR that was reduced by more than 25% from the baseline eGFR was defined as renal progression [18]. Each patients’ renal function was evaluated at the same medical center to reduce variability. Cigarette smoking was classified as smoking or never smoking. Smoking was defined as at least 100 cigarettes in a lifetime [19]. Alcohol consumption was classified as current alcohol consumption or never.

Because all of the participating research medical centers and communities used the same medical laboratory standards and protocols, research serum creatinine values from the different contributing research units can be compared and standardized with each other. This study was approved by the Joint Institutional Review Board of Tri-Service General Hospital (TSGHIRB 100-05-197), Kaohsiung Medical University Chung-Ho Memorial Hospital (KMUHIRB 20120019), Taipei Medical University (TMU-JIRB 20124036), National Cheng Kung University Hospital (A-ER-101-117), Kaohsiung Chang Gung Memorial Hospital (101-1096B), Cardinal Tien Hospital (TMU-JIRB 201204035), Changhua Christian Hospital (CCHIRB 120405), and China Medical University Hospital (DMR101-IRB2-273(CR-1). Written informed consent was obtained from the study participants before any data collection occurred.

### 2.3. Physical Activities Definitions

This study used the Taiwan version of the International Physical Activity Questionnaire (IPAQ), a short, last-seven-day self-administered format, to measure physical activity (http://www.ipaq.ki.se). The IPAQ is a validated instrument used to assess cross-national physical activity. It is a validated questionnaire that has been translated into multiple languages and has been extensively tested in many countries around the world [20,21,22]. The questionnaire collected information on the frequency of physical activity and measured vigorous-intensity activity, moderate-intensity activity, and walking activities in the past seven days. The IPAQ asks participants to report physical activities performed for more than 10 continuous minutes [23].

The metabolic equivalent of tasks (METs) represents the amount of oxygen consumed while sitting at rest and is equal to 3.5 mL/kg/min of VO2 Max [24]. METs were calculated based on the IPAQ questions in this study by assigning standard MET values for walking as well as moderate- and vigorous-intensity activity: 3.3 METs, 4.0 METs, and 8.0 METs, respectively (http://www.ipaq.ki.se). These values were used to calculate vigorous-intensity activity, moderate-intensity activity, and walking activity METs (minutes per week) as follows: walking activity METs = 3.3 × walking minutes × walking days; moderate-intensity activity METs = 4.0 × moderate-intensity activity minutes × moderate days; and vigorous activity METs = 8.0 × vigorous-intensity activity minutes × vigorous-intensity days. Total physical activity = sum of walking + moderate + vigorous MET minutes/week scores [23,25].

This study divided physical activity into three levels (low, moderate, and high) based on the following criteria: low physical activity was defined as not meeting the criteria of the moderate or high category; moderate physical activity was defined as meeting any of the following three conditions: (a) three or more days of vigorous-intensity activity of at least 20 min/day; (b) five or more days of moderate-intensity activity and/or walking of at least 30 min/day; or (c) five or more days of any combination of walking, moderate-intensity, or vigorous-intensity activities achieving at least 600 MET-minutes/week. High physical activity was defined as meeting any of the following two conditions: (a) vigorous-intensity activity on at least three days/week and accumulating at least 1500 MET-minutes/week; (b) seven or more days of any combination of walking, moderate-intensity, or vigorous-intensity activities achieving at least 3000 MET-minutes/week [23,25].

### 2.4. Statistical Analysis

The patient characteristics data were analyzed by descriptive statistics. The chi-square test and Student’s *t* test were used to determine the significant differences between patients with and without renal progression. The chi-square test and one-way analysis of variance test were used to determine the significant differences between levels of physical activity. The odds ratio (OR) and 95% confidence interval (CI) for the risk of renal progression were calculated for the levels of physical activity. After adjusting for all covariates, we used multivariate logistic regression models to evaluate the relationship between the level of physical activity and risk of renal progression. Covariates included sex, age, CKD, diabetes mellitus, dyslipidemia, stroke, gout, body mass index (BMI), serum creatinine, cigarette smoking, and alcohol consumption. We further performed a stratified analysis by sex and age to assess the association between levels of physical activity and renal progression. All analyses and calculations were performed using Statistical Analysis System (SAS) software (SAS Institute), version 9.4. The results were considered statistically significant at *p* < 0.05.

## 3. Results

A total of 30.29% of patients with hypertension revealed renal progression. The average follow-up time was 35.42 (SD ±16.13) months. Table 1 lists the baseline demographic and clinical characteristics of renal non-progression and renal progression among hypertensive patients. The mean age was 63.32 (SD ±12.29) years, and males accounted for 2043 (57.66%) of the hypertensive patients. The patients with renal progression had a higher prevalence of CKD (1016 (94.69%)), diabetes mellitus (573 (53.40%)), stroke (124 (11.56%)), and gout (409 (38.12%)), as well as lower levels of physical activity than those without renal progression.

Table 2 presents the baseline characteristics and level of physical activity of the study hypertensive patients. Among the 3543 patients in the overall cohort, 2235 (63.08%), 926 (26.14%), and 382 (10.78%) were in the low, moderate, and high level of physical activity, respectively. The patients in the low level of physical activity were older (63.29 (SD ±12.70)) than those in the moderate (64.19 [(SD ±11.33)) and high level (61.34 (SD ±11.86)). The low level of physical activity also had higher proportions of diabetes mellitus (1085 (48.55%)). Compared with the moderate and high level of physical activity, the low level of physical activity had a higher serum creatinine (0.73 (SD ±0.15)), BMI (25.98 (SD ±4.26)), and fasting glucose (116.90 (SD ±38.76)).

Table 3 shows the crude and adjusted odds ratio (OR) of the risk of renal progression across different levels of physical activity in hypertensive patients, calculated using the logistic regression model. The crude OR of renal progression was 1.67 (1.29–2.16) for low levels of physical activity and 1.48 (1.12–1.96) for moderate levels of physical activity compared with high levels of physical activity. After we adjusted for covariates, a higher risk of renal progression was still significantly associated with a low level of physical activity (OR, 1.39; 95% CI, 1.01–1.90) and a moderate level of physical activity (OR, 1.39; 95% CI, 1.04–1.86) compared with a high level of physical activity.

Table 4 lists the OR for the risk of renal progression across different levels of physical activity in hypertensive patients stratified by sex and age group. In males, the multivariate adjustment for age, CKD, diabetes mellitus, dyslipidemia, stroke, gout, BMI, serum creatinine, cigarette smoking, and alcohol consumption resulted in an OR for renal progression that was significantly associated with a low level of physical activity (OR, 1.56; 95% CI, 1.10–2.20) relative to the high level of physical activity (*p* for trend is <0.020). In females, there was no association between levels of physical activity and developing renal progression. In patients aged ≥60 years, after we adjusted for covariates, the resulting OR for renal progression was significantly associated with a low level of physical activity (OR, 1.54; 95% CI, 1.08–2.22) and a moderate level of physical activity (OR, 1.50; 95% CI, 1.02–2.21) relative to the high level of physical activity (*p* for trend is 0.052). The results for patients aged <60 years were not significant.

## 4. Discussion

This multicenter prospective cohort study enrolled subjects with hypertension to demonstrate that the level of physical activity was an independent predictor of renal progression. We found that a low level of physical activity and a moderate level of physical activity were associated with an increased risk of renal progression compared with a high level of physical activity. In particular, in male or elderly patients, different levels of physical activity were significantly associated with renal function. To our knowledge, this is the first prospective cohort study to elucidate the effects of physical activity differences on kidney damage in patients with hypertension.

Low levels of physical activity and low physical function are associated with adverse clinical outcomes [12]. A previous study indicated that higher levels of physical activity are attributed to a lower prevalence of CKD, which is broadly consistent with this study [26]. Insufficient physical activity is associated with increased morbidity and mortality, both in the general population and in patients with noncommunicable diseases [27,28]. Therefore, physical activity is related to primary prevention in the general population and the secondary and tertiary care of the patient population [29]. Many scientific studies indicate that most physiological systems can be positively altered by physical activity [30,31,32]. Participation also plays a role in the prevention and treatment of chronic diseases [33]. Therefore, physical activity could be a natural treatment for many diseases [34].

Our results indicated a trend toward men having a higher risk of renal progression with lower levels of physical activity than women. Previous studies have proposed that men show a lower risk of CKD with higher levels of vigorous intensity physical activity compared with women, while shorter sitting times are associated with CKD in women, but not in men [26]. A possible explanation for this finding is that cultural and social environmental differences and biological effects have caused sex differences [35,36,37]. It has been reported that estrogens may bring into play potent antioxidant action and may have protective effects on renal progression in women [38]. A previous study also shows sex steroids and sex chromosomes play a mediating role in causing kidney disease [39].

Our findings suggest that the level of physical activity is related to renal progression in older patients, but not in younger patients. These findings are consistent with those of previous studies that show that exercising with sufficient intensity and frequency can improve health in older adults [40,41]. The meta-analysis also reported that increased physical activity could prevent and slow down the onset of dysfunction and the progression of functional decline in the elderly [42]. However, with increasing age, the prevalence of physical exercise decreases [43]. In addition, our research shows that hypertensive patients with CKD, diabetes, stroke, or gout were associated with kidney function decline, which is consistent with previous studies. Diabetes mellitus, CKD, stroke, and gout are independent risk factors for renal disease [35,44,45,46].

Our study uses the IPAQ questionnaire to assess physical activity and calculate the level of physical activity (low, medium, and high) through MET. The IPAQ questionnaire has been validated and widely used in other studies [20,21,22,26]. MET is defined as the ratio of working metabolic rate to resting metabolic rate, and is usually used to indicate the intensity of physical activity [26]. According to the World Health Organization guidelines, four METs are assigned to the time spent doing moderate activities, and eight METs to the time spent doing vigorous activities [47].

Our study has advantages. First, our study provided evidence in a large cohort study showing that a low level of physical activity was an independent predictor of kidney function decline in patients with hypertension. Second, data on patient demographics, comorbidities, physical activity, and lifestyle were collected through face-to-face interviews. To ensure the quality of our data, our interviewers were given in-depth training. However, the authors acknowledge some limitations. First, voluntary participation in research may lead to selection bias. Second, misclassification of physical activity, which was recorded from the self-report questionnaires at baseline, is possible. However, this misclassification may be non-differential, thus leading to an underestimation of certain test results. Third, because the participants’ physical examination data were collected from multiple centers, equipment variations may affect the test results. Nonetheless, all supervisors tried to minimize measurement errors.

## 5. Conclusions

This study offers evidence demonstrating that physical activity is related to renal progression among patients with hypertension. We observed that a low level of physical activity had significant adverse effects, especially in male and elderly patients. Therefore, to reduce the risk of renal progression, we recommend that clinicians should encourage patients to improve physical activity. In addition, we recommend that healthcare providers develop strategies and clinical interventions to increase patients’ motivation for exercise.

## Figures and Tables

**Table 1:** The baseline characteristics subjects.

Characteristic	Overall	Renal Non-Progression	Renal Progression	*p*-Value
	(*n* = 3543)	(*n* = 2470)	(*n* = 1073)	
Sex, %				0.052
Female	1500 (42.34)	1019 (41.26)	481 (44.83)	
Male	2043 (57.66)	1451 (58.74)	592 (55.17)	
Age (years), mean ± SD	63.32 ± 12.29	63.02 ± 12.21	63.99 ± 12.45	0.031
Comorbidities, %				
CKD	2771 (78.21)	1755 (71.05)	1016 (94.69)	<0.001
Diabetes mellitus	1642 (46.34)	1069 (43.28)	573 (53.40)	<0.001
Dyslipidemia	1428 (40.30)	992 (40.16)	436 (40.63)	0.821
Stroke	330 (9.31)	206 (8.34)	124 (11.56)	0.003
Gout	1005 (28.37)	596 (24.13)	409 (38.12)	<0.001
Physical examination, mean ± SD				
Serum creatinine (mg/dL)	1.58 ± 1.22	1.29 ± 0.98	2.25 ± 1.44	<0.001
Baseline eGFR (mL/min per 1.73 m^2^)	52.70 ± 24.66	59.27 ± 22.43	37.57 ± 22.86	<0.001
Height (cm)	161.39 ± 8.28	161.71 ± 8.32	160.65 ± 8.12	<0.001
Weight (kg)	67.58 ± 12.91	68.03 ± 13.03	66.55 ± 12.58	0.002
BMI (kg/m^2^)	25.87 ± 4.11	25.93 ± 4.10	25.73 ± 4.14	0.181
Fasting glucose (mg/dL)	115.63 ± 36.33	113.54 ± 33.92	120.32 ± 40.87	<0.001
SBP (mmHg)	133.88 ± 16.70	133.07 ± 16.64	135.73 ± 16.70	<0.001
DBP (mmHg)	77.33 ± 11.61	77.63 ± 11.35	76.62 ± 12.17	0.022
Total cholesterol (mg/dL)	181.91 ± 38.58	181.45 ± 37.02	182.96 ± 41.88	0.332
Lifestyle, %				
Cigarette smoking	919 (25.95)	630 (25.51)	289 (26.96)	0.387
Alcohol consumption	423 (11.94)	304 (12.31)	119 (11.09)	0.332
Sitting Time (HR/per day), mean ± SD	242.84 ± 136.91	241.90 ± 137.36	244.53 ± 136.20	0.717
Levels of Physical Activity, %				<0.001
Low	2235 (63.08)	1519 (61.50)	716 (66.73)	
Moderate	926 (26.14)	653 (26.44)	273 (25.44)	
High	382 (10.78)	298 (12.06)	84 (7.83)	

Data expressed as percentage or mean ± SD. Abbreviations: CKD, chronic kidney disease; BMI, body mass index; SBP, systolic blood pressure; DBP, diastolic blood pressure; HR, heart rate; eGFR, estimated glomerular filtration rate.

**Table 2:** Levels of physical activity by patient characteristics.

Characteristic	Low	Moderate	High	*p*-Value
	*n* = 2235	*n* = 926	*n* = 382	
Sex, %				<0.001
Female	1021 (45.68)	380 (41.04)	99 (25.92)	
Male	1214 (54.32)	546 (58.96)	283 (74.08)	
Age (years), mean ± SD	63.29 ± 12.70	64.19 ± 11.33	61.34 ± 11.86	<0.001
Comorbidities, %				
CKD	1765 (78.97)	725 (78.29)	281 (73.56)	0.061
Diabetes mellitus	1085 (48.55)	404 (43.63)	153 (40.05)	0.001
Dyslipidemia	881 (39.42)	375 (40.50)	172 (45.03)	0.118
Stroke	214 (9.57)	83 (8.96)	33 (8.64)	0.771
Gout	619 (27.70)	262 (28.29)	124 (32.46)	0.161
Physical examination, mean ± SD				
Serum creatinine (mg/dL)	1.63 ± 1.28	1.52 ± 1.16	1.46 ± 1.02	0.010
Baseline eGFR (mL/min per 1.73 m^2^)	51.89 ± 25.26	53.31 ± 23.77	55.90 ± 22.97	0.009
Height (cm)	161.14 ± 8.34	161.23 ± 8.17	163.16 ± 7.96	<0.001
Weight (kg)	67.66 ± 13.31	66.64 ± 11.87	69.37 ± 12.84	0.002
BMI (kg/m^2^)	25.98 ± 4.26	25.58 ± 3.85	25.96 ± 3.80	0.044
Fasting glucose (mg/dL)	116.90 ± 38.76	114.31 ± 32.27	111.53 ± 30.44	0.016
SBP (mmHg)	133.94 ± 16.82	133.81 ± 16.63	133.67 ± 16.18	0.948
DBP (mmHg)	77.18 ± 11.35	77.61 ± 12.22	77.51 ± 11.58	0.609
Total cholesterol (mg/dL)	181.97 ± 39.65	182.32 ± 38.26	180.59 ± 32.74	0.777
Lifestyle, %				
Cigarette smoking	561 (25.10)	237 (25.62)	121 (31.68)	0.025
Alcohol consumption	261 (11.68)	102 (11.02)	60 (15.71)	0.048
Sitting time, mean ± SD	246.43 ± 139.73	240.75 ± 135.73	232.71 ± 127.43	0.396

Abbreviations: CKD, chronic kidney disease; BMI, body mass index; SBP, systolic blood pressure; DBP, diastolic blood pressure.

**Table 3:** Risk of renal progression across different levels of physical activity.

Variable	Crude Odds Ratio (95% CI)	Adjusted Odds Ratio (95% CI)
Levels of Physical Activity		
High	Reference	Reference
Moderate	1.48 (1.12–1.96)	1.39 (1.01–1.90)
Low	1.67 (1.29–2.16)	1.39 (1.04–1.86)
Sex		
Male	Reference	Reference
Female	1.16 (1.00–1.33)	1.16 (1.00–1.33)
Age (years)	1.01 (1.00–1.01)	1.00 (0.99–1.00)
Comorbidities, %		
CKD	7.26 (5.48–9.61)	3.87 (2.86–5.24)
Diabetes mellitus	1.50 (1.30–1.73)	1.75 (1.48–2.08)
Dyslipidemia	1.02 (0.88–1.18)	1.01 (0.85–1.19)
Stroke	1.44 (1.14–1.82)	1.30 (1.00–1.70)
Gout	1.94 (1.66–2.26)	1.47 (1.23–1.77)
BMI (kg/m^2^)	0.99 (0.97–1.01)	0.99 (0.97–1.01)
Serum creatinine (mg/dL)	2.05 (1.90–2.21)	2.00 (1.83–2.18)
Cigarette smoking	1.08 (0.92–1.27)	1.09 (0.88–1.35)
Alcohol consumption	0.87 (0.71–1.11)	0.98 (0.76–1.28)

Abbreviations: CI, confidence interval; CKD, chronic kidney disease; BMI, body mass index; SBP, systolic blood pressure; DBP, diastolic blood pressure; OR, odds ratio.

**Table 4:** Risk of renal progression across different levels of physical activity by gender and age.

Subgroups	High	Moderate Odds Ratio (95% CI)	Low Odds Ratio (95% CI)	*p* for Trend
Male	Reference	1.45 (0.99–2.12)	1.56 (1.10–2.20)	<0.020
Female	Reference	1.20 (0.63–1.87)	1.09 (0.67–2.12)	0.866
Age ≥ 60	Reference	1.50 (1.02–2.21)	1.54 (1.08–2.22)	0.052
Age < 60	Reference	1.52 (0.87–2.64)	1.51 (0.92–2.48)	0.191

Reference group is high. Odds ratio were adjusted for covariate factors, including age, CKD, diabetes mellitus, dyslipidemia, stroke, gout, BMI, serum creatinine, cigarette smoking, and alcohol consumption.
